# Pain Control for Sickle Cell Crisis, a Novel Approach? A Retrospective Study

**DOI:** 10.3390/medicina59122196

**Published:** 2023-12-18

**Authors:** Amélie Rollé, Elsa Vidal, Pierre Laguette, Yohann Garnier, Delphine Delta, Frédéric Martino, Patrick Portecop, Maryse Etienne-Julan, Pascale Piednoir, Audrey De Jong, Marc Romana, Emmanuelle Bernit

**Affiliations:** 1 Anesthesiology and Intensive Care Department, University Hospital of La Guadeloupe, F-97139 Les Abymes, France; elsa.vidal@chu-guadeloupe.fr (E.V.); pierre.laguette@chu-guadeloupe.fr (P.L.); frederic.martino@chu-guadeloupe.fr (F.M.); pascale.piednoir@chu-guadeloupe.fr (P.P.); 2 Université Paris Cité and Université des Antilles, INSERM, BIGR, F-75015 Paris, France; yohann.garnier@inserm.fr (Y.G.); maryse.etienne-julan@chu-guadeloupe.fr (M.E.-J.); marc.romana@inserm.fr (M.R.); 3 West-Indies Faculty of Medicine, University of The French West-Indies, F-97157 Pointe à Pitre, France; delphine.delta@chu-guadeloupe.fr; 4 Emergency Department, University Hospital of Guadeloupe, F-97100 Pointe à Pitre, France; patrick.portecop@chu-guadeloupe.fr; 5 Sickle Cell Disease Unit, Reference Centre for Sickle Cell Disease, Thalassemia and Other Red Cell Rare Diseases, CHU de la Guadeloupe, CEDEX, F-97159 Pointe à Pitre, France; emmanuelle.bernit@chu-guadeloupe.fr; 6 Anesthesia and Critical Care Department, Saint Eloi Teaching Hospital, University Montpellier 1, 80 Avenue Augustin Fliche, CEDEX 5, F-34295 Montpellier, France; a-de_jong@chu-montpellier.fr; 7 Phymed Exp INSERM U1046, CNRS UMR 9214, F-34295 Montpellier, France

**Keywords:** acute pain and peri-operative medicine, emergency pain, anatomic and techniques, chronic pain, sickle cell disease, vaso-occlusive crisis

## Abstract

*Background and Objectives*: Pain management poses a significant challenge for patients experiencing vaso-occlusive crisis (VOC) in sickle cell disease (SCD). While opioid therapy is highly effective, its efficacy can be impeded by undesirable side effects. Local regional anesthesia (LRA), involving the deposition of a perineural anesthetic, provides a nociceptive blockade, local vasodilation and reduces the inflammatory response. However, the effectiveness of this therapeutic approach for VOC in SCD patients has been rarely reported up to now. The objective of this study was to assess the effectiveness of a single-shot local regional anesthesia (LRA) in reducing pain and consequently enhancing the management of severe vaso-occlusive crisis (VOC) in adults with sickle cell disease (SCD) unresponsive to conventional analgesic therapy. *Materials and Methods*: We first collected consecutive episodes of VOC in critical care (ICU and emergency room) for six months in 2022 in a French University hospital with a large population of sickle cell patients in the West Indies population. We also performed a systematic review of the use of LRA in SCD. The primary outcome was defined using a numeric pain score (NPS) and/or percentage of change in opioid use. *Results*: We enrolled nine SCD adults (28 years old, 4 females) for ten episodes of VOC in whom LRA was used for pain management. Opioid reduction within the first 24 h post block was −75% (50 to 96%). Similarly, the NPS decreased from 9/10 pre-block to 0–1/10 post-block. Five studies, including one case series with three patients and four case reports, employed peripheral nerve blocks for regional anesthesia. In general, local regional anesthesia (LRA) exhibited a reduction in pain and symptoms, along with a decrease in opioid consumption post-procedure. *Conclusions*: LRA improves pain scores, reduces opioid consumption in SCD patients with refractory pain, and may mitigate opioid-related side effects while facilitating the transition to oral analgesics. Furthermore, LRA is a safe and effective procedure.

## 1. Introduction

Sickle cell disease (SCD) is the most common genetic disorder in the world caused by the production of abnormal hemoglobin (Hb), the so-called hemoglobin S (HbS) [1]. Several genotypes lead to SCD, including homozygosity for the sickle hemoglobin (HbS) gene (i.e., a missense mutation [Glu6Val, rs334] in the β-globin gene [HBB]) and various compound heterozygous states including HbSC or HbSb-thalassemia [2]. At a deoxygenated state, the abnormal HbS forms rigid polymers. Such HbS polymerization, promoted by various conditions such as hypoxia, cold, or infections, leads to the sickling of red blood cells (RBCs). These brittle and rigid sickle-shaped red blood cells (SS-RBCs) are unstable and prone to hemolysis and occlude microcirculation, causing vaso-occlusion, downstream tissue ischemia associated with pain, and ultimately end-organ damages [3].

In addition to these abnormalities, other key cellular actors, such as activated vascular endothelium, adherent reticulocytes, activated neutrophils, monocytes and platelets, and mastocytes, are involved in the pathophysiology of SCD [4,5]. Chronic hemolytic anemia leads to a decrease in oxygen-carrying capacity and tissue hypoxia. Through its effects on vascular function, inflammation, and oxidative stress [6] partly related to the release in the circulation of hemoglobin and heme, two well-characterized damaged-associated molecular patterns, chronic hemolysis may play a role in progressive multi-organ damage, such as cerebral vasculopathy, pulmonary hypertension, kidney disease, leg ulcers, and priapism [7].

SCD pain pathophysiology is multifactorial, involving multiple molecular and cellular partners. More recently, peripheral and central neurologic involvement inducing neurogenic inflammation and inadequate response of the autonomic nervous system has been shown to be implicated in this pathophysiological condition and could partly explain the resistance of pain to common opioid treatments. Thus, there are numerous barriers to effective management, making treatment of acute painful sickle crises extremely challenging [8,9]. Frequent or intense painful vaso-occlusive crises (VOC) are associated with the occurrence of severe complications of the disease, such as acute chest syndrome, acute multi-organ failure, or death [2]. Thus, early and optimal management of pain is required in these patients.

Opioids continue to be the primary treatment for acute pain episodes, albeit not without adverse effects. Medications such as morphine, hydromorphone, and fentanyl are commonly used for this purpose [10,11]. Intravenous therapy with scheduled or continuous dosing through patient-controlled analgesia is recommended for SCD patients admitted for pain management [12,13].

In addition to the well-documented adverse effects associated with opioids, the management of acute painful crises has seen limited changes, and the prevention and treatment of vaso-occlusive crises (VOC) remain suboptimal. Despite advancements in understanding the pathophysiology of pain and the pharmacogenomics of opioids, these insights have not translated effectively into the management of VOC in SCD [3]. Indeed, numerous challenges persist with the frequent use of opioid therapy, particularly in relation to opioid tolerance and opioid-induced hyperalgesia triggered by N-methyl-D-aspartate (NMDA) receptor activation [14]. Tolerance leads to escalating dosage requirements over time, while hyperalgesia may necessitate tapering opioids and a shift in the therapeutic approach [14].

Some alternative therapeutics exist, like ketamine or magnesium, which are both noncompetitive antagonists of NMDA receptors [15]. Moreover, magnesium has vasodilator activity and exhibits anti-inflammatory properties [16,17,18,19]. Medical trials are currently acting to integrate those strategies as a bundle of pain plans.

More and more frequently, clinicians explore the efficiency of non-intravenous opioid treatment to improve the treatment of acute pain in SCD patients. Moreover, they advocate management procedures based on the pathophysiology mechanisms of sickle cell pain and a personalized strategy, as this disease is characterized by high individual phenotypic variation.

In addition to the management of vaso-occlusive crisis in patients with sickle cell disease, it is noteworthy that anesthesia techniques have played a crucial role in addressing pain, particularly in the context of cancer. For instance, in 10–20% of patients with cancer pain where standard treatment is not effective, anesthesia techniques such as epidural, subarachnoid, intrathecal, and peripheral nerve blocks have demonstrated efficacy. These techniques allow for the administration of opioids together with local anesthetics on time, as required, or continuously [20,21,22,23].

Our study proposes a novel approach for the management of VOC, the so-called local regional anesthesia (LRA). LRA has many targets and may have a key role in the following fields. LRA is traditionally used for its antinociceptive effects because of its ability to block Na+ channels [24]. In addition, LRA interacts with other cellular systems, such as the inflammatory system, known to be a key player in the genesis of VOC [24]. Indeed, LRA inhibits local neurogenic inflammation and, therefore, the phenomena of sensitization, hyperalgesia, and chronic pain, and is presently described as an anti-inflammatory treatment [24]. Moreover, LRA influences vasodilation in limbs and has a beneficial impact on tissue oxygenation. For example, Tighe et al. demonstrated sustained increases in tissue rSO_2_ values following LRA [25]. However, side effects could occur (incapacitating block motor, paresthesia, and local anesthetic systemic toxicity (LAST)) [26].

In this study, our objectives were: (1) to assess the efficacy of a single-shot local regional anesthesia (LRA) in effectively reducing pain and improving the management of severe vaso-occlusive crisis (VOC) in adults with SCD unresponsive to conventional analgesic therapy, (2) to evaluate the safety of the LRA procedure, and (3) to conduct a systematic review in pursuit of our research goals.

## 2. Materials and Methods

### 2.1. Data Collection

Between May and December 2022, we collected consecutive episodes of VOC in critical care units (intensive care unit (ICU) and emergency room). This retrospective analysis of prospectively collected data was performed in the French West Indies University Hospital of Guadeloupe. We enrolled SCD adults hospitalized for episodes of VOC in whom LRA was used for pain management because of refractory pain despite multimodal therapy. The present study was conducted in accordance with the Declaration of Helsinki and approved by the Ethics Committee of the University Hospital of La Guadeloupe (protocol code “A116_10072023” and “07102023”, approved on 10 July 2023). 

Data were anonymized prior to statistical analysis. All patient consent was obtained.

The co-primary outcome was defined by the numerical pain scores (NPS); the percentage change of morphine consumption 24 h before and 24 h after LRA (%change = [(pre-block use) − (post-block use)]/(pre-block use) × 100). The numerical pain scale (NPS) ranges from 0 (no pain) to 10 (worst possible pain). Additionally, we gathered clinical features related to vaso-occlusive crises (VOC), including the pain site, location of the perineural block, duration of block sensitivity, average pre-pain intensity, post-pain intensity on the NPS, and 24 h morphine equivalent consumption both before and after local regional anesthesia (LRA) administration. Average pain scores were computed by determining the mean of five or six NPS scores (within a 6 h window) before LRA injection and five or six NPS scores (within a 6 h window) after LRA injection.

We also collected side effects such as incapacitating block motor, paresthesia, and LAST occurring during the post-procedure hospital stay. The LRA satisfaction was evaluated using a Likert scale ranging from 0 (not satisfied) to 10 (very satisfied).

All patients underwent the same multimodal analgesia protocol. The pre-treatment to post-treatment morphine equivalents were calculated by converting the usage of peripheral opioids 24 h before and 24 h after the local regional anesthesia (LRA) injection.

#### 2.1.1. Characteristics of the LRA Procedures

After emergency admission for hyperalgesia (NPS > 6 more than 6 h) or opioid refractory VOC, the patient was taken to the post-anesthesia care unit (PACU), where he was scoped by close monitoring (saturation, respiratory rate, heart rate, and blood pressure) before and after the LRA procedure under oxygen therapy (by nasal cannula). If the SCD patient was previously in ICU, the procedure and the monitoring were applied in his own unit. An attending anesthesiologist realized all the procedures. Under direct visualization using a high-frequency linear ultrasound transducer, a 22 G echogenic needle was perineurally inserted in-plane. Ropivacaine (1–2 mg/kg) was deposited perineurally with clonidine as an adjuvant to 1 μg/kg. The dosing recommendations for ultrasound-guided peripheral regional anesthesia, falling within 0.5–1.5 mg/kg as advised by the American and European Society of Regional Anesthesia [27], were adhered to. The maximum described toxic dose of ropivacaine is 3.0 mg/kg [28]. The appropriate spread of volume in specific peripheral blocks is crucial. Following this principle, we utilized ropivacaine 0.2%. Moreover, employing lower concentrations with larger volumes can help address dosing challenges and mitigate the risk of motor block [29].

#### 2.1.2. Statistical Methods

We employed descriptive statistics to characterize categorical variables, reporting a mean ± SD or median ± interquartile range (IQR) for continuous variables.

Categorical outcomes were reported with numbers and percentages. For each episode, we first calculated the percentage change of morphine consumption 24 h before and 24 h after LRA (%change = [(pre-block use) − (post-block use)]/(pre-block use) × 100), and then we estimated the median and the interquartile range for all episodes. All analyses were performed using R 3.4.4 (R Project, Vienna, Austria).

### 2.2. The Review: Search Strategy for the Systematic Review

We conducted a computerized search on EMBASE and the Cochrane Center Register of Controlled Trials (CENTRAL) for studies related to local regional anesthesia (LRA) in the treatment of VOC in SCD patients (from 1988 to 31 December 2022). Only English publications were included. In our bibliographic review, we used the keywords (“Local regional anesthesia” OR “peripheral nerve block” OR “Sickle cell disease” OR “SCD”) in our Boolean search strategy. Additionally, we examined references in the retrieved articles for relevant publications. Any duplicate papers were identified and removed. All potential eligible papers underwent a full retrieval. For the systematic review, data were extracted as reported in the original papers, and individual pain trajectory data were described for each study.

## 3. Results

### 3.1. The Case Series: Data Collection

**Figure 1:**
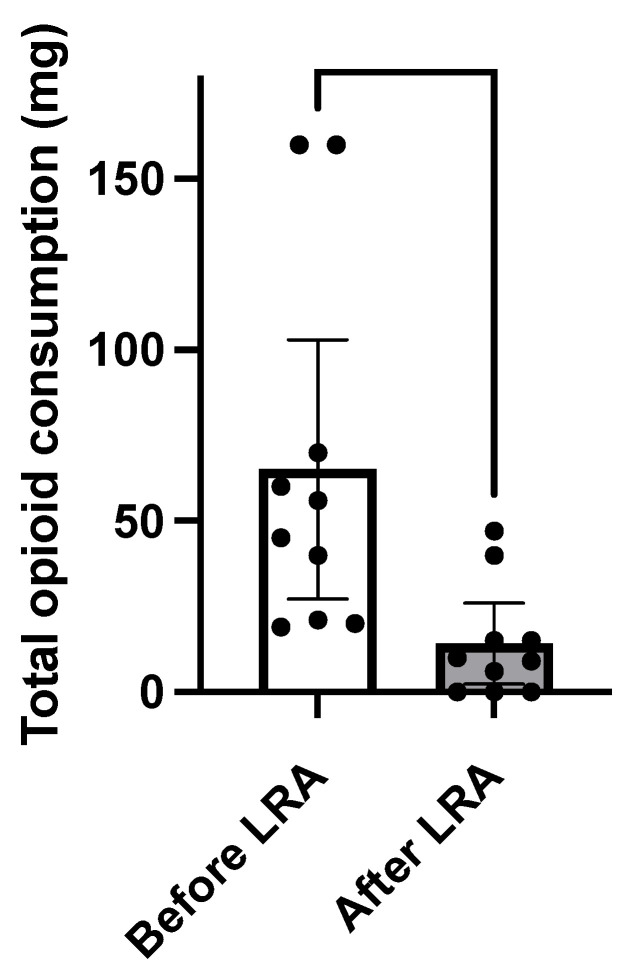
Comparison of opioid consumption before and after LRA. Local regional anesthesia (LRA) was effective in treating sickle cell crises for the reductions in opioid consumption. Opioid reduction within the first 24 h post-block was −75% (50 to 96%, *p* = 0.016, *). The percentage change of morphine consumption 24 h before and 24 h after LRA (%change = [(pre-block use) − (post-block use)]/(pre-block use) × 100. Then, we performed a paired *t*-test (*p*-value).

**Figure 2:**
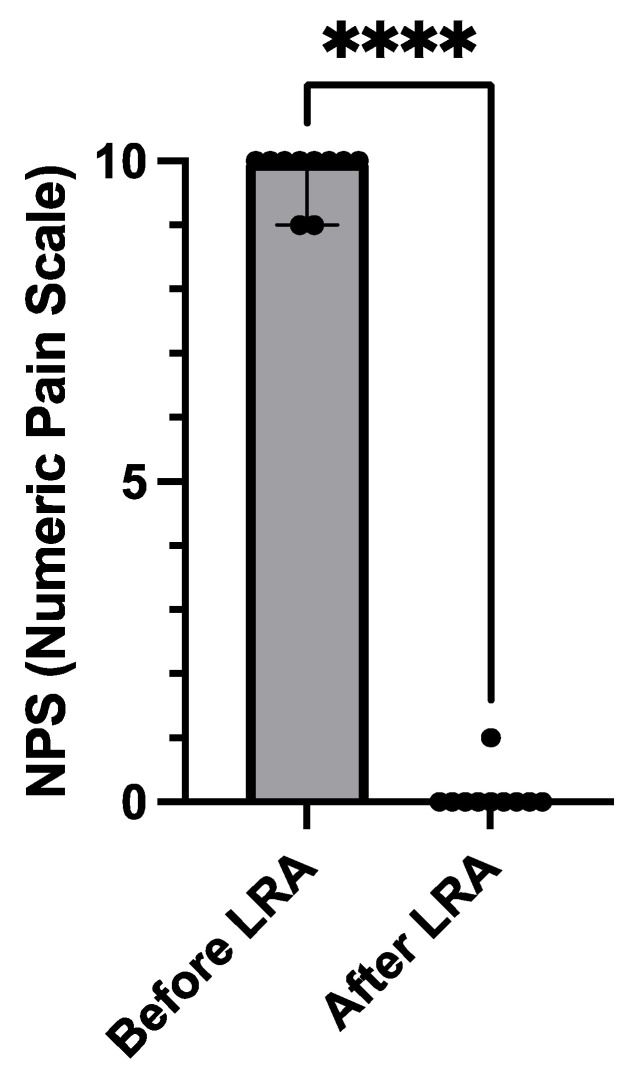
Comparison of numeric pain scale before and after LRA. Local regional anesthesia (LRA) was effective in treating sickle cell crises for the reduction in pain trajectory. Numeric pain scale (NPS) decreased from 9/10 pre-block to 0–1/10 post-block (less than 6 h later) (*p* < 0.001, ****).

Nine adult SCD patients (28 years old [22,23,24,25,26,27,28,29,30,31,32], four females, and seven HbSS and two HbSC) were treated for ten episodes with LRA for refractory pain despite multimodal therapy. The length of stay with opioid refractory VOC was 11 h (ranging from 6 to 39 h) in the emergency unit before the LRA procedure was undertaken. Pain scores and opioid consumption decreased within 24 h after LRA injection. Opioid reduction within the first 24 h post-block was −75% (95%CI, 50 to 96%, *p* = 0.016) (Table 1 and Figure 1). Similarly, NPS decreased from 9/10 pre-block to 0–1/10 post-block (*p* < 0.001) (Figure 2).

The analgesic effect was quickly effective (pain score 0 to 1) in all SCD patients in our cohort. The block duration of analgesia was 12 to 16 h; multimodal analgesia was sufficient for the relay, or at least reinjection was needed for five episodes (50%) (Table 2 and Supplemental Figure S1). One patient developed paresthesia; in another, a temporarily incapacitating block motor occurred, and no LAST was reported. There were no other major complications, and LRA was not associated with sedation, respiratory depression, or toxicity. Three patients experienced IV opioid-related adverse effects (hyperalgesia, hallucinations, and excessive sedation) and reported it themselves.

### 3.2. The Review

We identified 35 articles using our search strategy. After excluding articles where epidural anesthesia was employed, we included five studies (one case series with three patients and four case reports). Overall, local regional anesthesia (LRA) demonstrated a reduction in pain and symptoms, as well as decreased opioid consumption post-procedure in children (see Table 3). The most common medications used were bupivacaine and ropivacaine, with or without adjuvants such as dexmedetomidine. The majority of patients receiving LRA for vaso-occlusive crisis (VOC) were children. No significant adverse outcomes, such as cardio-respiratory arrest, anaphylaxis, or toxicity, were reported due to the use of LRA. One randomized controlled trial (RESCUE Phase 1) involved an emergency department physician performing nerve blocks as a phase one trial to assess feasibility. However, this study was terminated early due to a lack of resources. Nevertheless, our study is the first to report data collected for the LRA procedure using a single shot of perineural analgesic injection as a treatment for opioid-refractory VOC in adult SCD patients.

## 4. Discussion

For the first time, to our best knowledge, our study evaluated a cohort of nine SCD adult patients who were consecutively hospitalized for opioid-refractory VOC episodes. Our data suggest that LRA is effective for the reductions in pain trajectory (NPS decreased from 9/10 pre-block to 0–1/10 post-block, *p* < 0.001) and opioid consumption (−75% (50 to 96%, IC95), *p* < 0.016) and safe. Similarly, our search review highlights a significant decrease in morphine consumption and pain score for all case reports included, which are one of the current research investigation aims of acute VOC management in SCD patients in emergency departments.

Episodes of acute pain emerge as the defining characteristic of sickle cell disease (SCD) and persist as the leading cause of hospitalization for individuals with SCD [35]. Navigating this pain presents challenges due to the limited array of available treatment modalities [36]. Despite pain being a universal aspect for those with SCD, they remain among the most undertreated populations [37]. The standard protocol for managing painful episodes centers on rest, rehydration, oxygenation, and the use of analgesics such as acetaminophen, oral and parenteral non-steroidal anti-inflammatory drugs, as well as oral, parenteral, or continuous infusion of opioids [38]. The etiology of SCD vaso-occlusive crisis (VOC) is complex and is associated with nociceptive, neuropathic, autonomic, and inflammatory-mediated receptors [3,39].

Because of the multitarget actions of LRA, we believe that we should consider this technique in the treatment of such acute pain mechanisms and that it might be a part of the goal of individualized pain plans. It is worth noticing that opioid therapies carry numerous undesirable side effects, most notably sedation and respiratory depression. In our study, 30% of our patients experienced IV opioid-related adverse effects. In addition, LRA could present additional therapeutic effects. In surgical patients, local regional anesthesia (LRA) attenuates autonomic nociception and the inflammatory response in comparison to opioids, thereby alleviating constipation and opioid-induced hyperalgesia [40].

Furthermore, LRA promotes vasodilation, as demonstrated in a prospective analysis using near-infrared spectroscopy [25]. Such a vasodilator effect of LRA improves regional blood flow to ischemic areas and so reduces sickling [25].

The adoption of the local regional anesthesia (LRA) approach has been limited, in part, due to the unfamiliarity of this procedure among most hematologists. Nevertheless, there is a clinical necessity to curtail systemic opioid exposure [41]. Prolonged and recurrent pain crises expose patients to risks such as opioid tolerance, dependence, hyperalgesia, and chronic pain [39]. The incorporation of LRA in our subset of patients experiencing a vaso-occlusive crisis (VOC) resulted in a decreased need for opioids, improved pain relief, reduced hospitalization duration, and enhanced physical rehabilitation, contributing to higher patient satisfaction (see Table 3).

Retrospective case reports have also demonstrated favorable outcomes with a reduced need for opioids, shorter hospitalization durations, and enhanced patient satisfaction in cases of isolated limb vaso-occlusive crisis (VOC) [42]. Although there are no randomized clinical trials investigating the long-term effectiveness and safety of local regional anesthesia (LRA) in VOC, the available data strongly advocate for the inclusion of LRA in the treatment of painful VOC. It is noteworthy that the 2020 guidelines from the American Society of Hematology (ASH) for the management of acute and chronic pain in sickle cell disease (SCD) recommend interdisciplinary and multimodal approaches for pain treatment [43].

Two out of nine (22%) SCD cohort patients described a hyperalgesia phenomenon. They clearly described a sustained increase in pain when morphine was administrated. After LRA, they were the most satisfied patients we have seen so far. It is so-called opioid-induced hyperalgesia [40]. In contrast to opioids, local regional anesthesia (LRA) may circumvent these issues by employing a more direct approach to central nervous system receptors (and gate control), thereby avoiding hyperalgesia [44].

In our patient cohort, we deliberately opted for a lower concentration of ropivacaine (0.2%), leading to enhanced pain control while minimizing motor impairment, enabling early ambulation, and facilitating physical therapy. The beneficial effects of LRA in the treatment of localized refractory pain during VOC in the SCD population seem to be multiple: (i) it improves pain control, (ii) it decreases opioid usage, (iii) it reduces inflammation, (iv) it reduces HbS polymerization and adhesive events via vasodilation, and (v) it improves oxygenation. Our study strongly suggests that LRA could be a part of clinical decision-making options, considering an individualized approach and appropriately dosed local anesthetics to facilitate sensory blockade with the preservation of motor and physical function. Nevertheless, the sole study to date, which sought to assess the feasibility of single-shot femoral nerve blocks in patients admitted to the emergency department with acute pain crises involving the lower extremities, was prematurely terminated due to resources [45].

Among the limitations of our study, we focused on local regional anesthesia (LRA) exclusively for sickle cell disease (SCD) patients unresponsive to opioids. Additionally, one patient experienced post-LRA paresthesia, similar to a case reported by Giabicani et al., where peripheral neuropathy occurred after a popliteal sciatic nerve block in a patient with SCD [46]. Nerve injury is a well-recognized complication of LRA. Capdevila et al. reported an incidence of 0.21% [47], the main mechanism described so far being intraneural injection or direct nerve injuries [48]. In addition to adverse factors related to the LRA technique [49], other causes, such as concomitant patient disease (e.g., pre-existing subclinical polyneuropathy [50]) and the neurotoxicity of local anesthetics [26], could also be involved. Notably, recent data suggest an underestimation of neuropathy SCD-related diagnosis [51]. Further studies are warranted to determine the prevalence, physiopathology, and preventive treatment of SCD’s neuropathy.

## 5. Conclusions

Within our cohort, local regional anesthesia proved effective and safe in treating sickle cell crises, resulting in reduced pain trajectories and opioid consumption. Early implementation of this technique in the treatment of painful crises may act as a protective factor by breaking the cycle of vaso-occlusive crisis (VOC). Sickle cell disease (SCD) patients experiencing isolated limb crises are particularly suitable candidates for local regional anesthesia (LRA). Moreover, we intend to explore the feasibility of the LRA procedure as a primary modality for VOC and as an integral component of personalized pain management plans. Further investigations into the underlying physiological mechanisms responsible for the beneficial effects of LRA in VOC are warranted and should be conducted. 

## Figures and Tables

**Table 1:** Demographic characteristics of adult sickle cell disease patients receiving local regional anesthesia for improved pain management in severe VOC unresponsive to conventional analgesics.

Case	Indications	Block	% Change in Opioid Use	NPS Before/After	Opioid or Ketamine Related Events
Case 1, E1: 32-year-old male with HbSC (BMI:18)	Unilateral left upper extremity pain	Left Axillary Brachial Plexus Block	−96.3%	9/0	Hyperalgesia
Case 1, E2: 32-year-old male with HbSC (BMI:18)	Unilateral left lower limb pain	Left Femoral nerve block and Popliteal Sciatic Nerve Block	−32.9%	10/0	-
Case 2: 21-year-old female with HbSS (BMI:18)	Unilateral left lower limb pain	Left Popliteal Sciatic Nerve Block	−100%	10/0	-
Case 3: 32-year-old male with HbSS (BMI:20)	Bilateral shoulder pain	Femoral left and right Nerve Block	−100%	9/0	-
Case 4: 33-year-old male with HbSS (BMI:20)	Bilateral upper-extremity pain	Popliteal Sciatic Nerve Block	−90.6%	10/0	Sedation
Case 5: 21-year-old female with HbSC (BMI:18)	Unilateral left lower limb pain	Left Popliteal Sciatic Nerve Block	−100%	10/0	-
Case 6: 22-year-old female with HbSS (BMI:23)	Bilateral lower limb pain	Bilateral Femoral and Popliteal Sciatic Nerve Block	−21.1%	10/1	-
Case 7: 24-year-old male with HbSS (BMI:20)	Unilateral left upper extremity pain	Left Axillary Brachial Plexus Block	−50%	10/0	-
Case 8: 23-year-old male with HbSS (BMI:18)	Low back pain and pelvic pain	Transversus-abdominis pain (TAP) block	−75%	10/0	Hyperalgesia Hallucination Sedation
Case 9: 39-year-old female with HbSS (BMI:20)	Unilateral left lower limb pain	Left Femoral and Popliteal Sciatic Nerve Block	−55%	10/0	-

Percentage of change in opioid use, comparing 24 h prior to block and 24 h after block: %change = [(pre-block use) − (post-block use)]/(pre-block use) × 100%, NPS numeric pain scale, BMI body mass index.

**Table 2:** General patient data and follow-up in adult sickle cell disease patients receiving local regional anesthesia for enhanced pain management in severe VOC unresponsive to conventional analgesics: initial clinical and biological insights.

	All Patients (*n* = 9) Episodes (*n* = 10)
**Baseline characteristics**	
Age (Years)	28 (22–32)
Hemoglobin (g/dL) at steady state	9 (7.5–9)
LDH (UI/L) at steady state	425 (266–539)
**Clinical Presentation**	
Onset of symptoms to hospitalization (Days)	1 (0–2.5)
Systolic blood pressure (mmHg)	113 (101–122)
Mean blood pressure (mmHg)	73 (67–81)
Heart rate (/min)	103 (90–115)
Heart rate > 110/min	4 (40)
Respiratory rate (/min)	21 (19–25)
Transcutaneous saturation O_2_ (%)	100 (97–100)
Temperature (°C)	37 (37–37)
VOC reason for admission	9 (90)
VOC number of site(s)	1 (1–2)
Numeric pain scale (points)	10 (10–10)
**Biological presentation at hospitalization onset**	
Hemoglobin (g/dL)	8.3 (7.8–9.7)
LDH (UI/L)	498 (390–758)
Clinical evolution	
Developing secondary ACS	0 (0)
Sepsis	2 (20)
Shock	0 (0)
**Local Regional Anesthesia procedure details**	
Length between emergency stay and LRA (days)	1 (0.3–1)
Number of nerves blocked by episodes	2 (1–3)
Number of nerves blocked during the study time	14
LRA reinjection during the study time	5 (50)
Total of ropivacaine perineural injection (mg)	80 (50–80)
Total of perineural volume injection	40 (20–40)
Patient satisfaction with LRA	9 (10)
Paresthesia	1 (10)
**Outcome**	
Length of emergency stay (hours)	11 (6–39)
Length of ICU stay (days)	5.5 (2.5–8)
Length of hospitalization stay (days)	5.5 (2.7–9)
Transfusion	2 (20)
Numbers of transfusion	2 (2–2)

Data are expressed in median and interquartile 25–75 or number and percentage.

**Table 3:** Systematic review on the efficacy of local regional anesthesia in alleviating pain and enhancing the management of severe VOC in adults with sickle cell disease unresponsive to conventional analgesic therapy.

Study	Indications	Block	% Change in Opioid Use	NPS Before/After	Opioid or Ketamine Related Events
Karsenty, Pediatric Blood Cancer, 2022 [30] 16 year-old female with HbSS	Left upper-extremity pain (AVN of left humeral head)	Left supraclavicular nerve block catheter Ropivacaine	−78.7%	NA	Constipation sedation pruritis
Karsenty, Pediatric Blood Cancer, 2022 [30] 13 year-old male with HbSS	Left shoulder pain (History of AVN)	Left Interscalene nerve block catheter, Ropivacaine and dexmedetomine (4 μg)	−47.5%	NA	Constipation sedation
Karsenty, Pediatric Blood Cancer, 2022 [30] 11 year-old male with HbSC	Right upper-extremity pain (AVN of right humeral head)	Right interscalene nerve block catheter Ropivacaine	−79.6%	NA	Constipation
Weber, A & A Case Reports, 2017 [31] 14 year-old male HbSS	Right Lower extremity (Ankle)	Right popliteal sciatic nerve block catheter Ropivacaine	−29.8%	10/0 to 2	Opioid related hypoxia
Wyatt, Journal of Clinical Anesthesia, 2020 [32] 15 year-old male (SCD status not reported)	Right Lower extremity (Hip and thigh)	Right Pericapsular nerve group (PENG) and femoral nerve (FN) block Bupivacaine and dexmedetomine	−89.2%	10/0	Sedation
Hasan, A & A Practice, 2019 [33] 10 year-old male (SCD status not reported)	Right upper quadrant pain (Acute cholecystitis)	A single-shot thoracic paravertebral nerve block (PVB) and rectus sheath blocks Ropivacaine	NA	10/0 to 1	-
Vuong, Open Journal of Anesthesia, 2012 [34] 12 year-old female with HbSS	Right lower extremity (Severe thigh pain)	Bilateral Femoral and Popliteal Sciatic nerve blocks via catheter Ropivacaine	NA	10/1	-

## Data Availability

Data supporting reported results can be shared. Please contact the corresponding author (amelie.rolle.mcu@gmail.com).

## Supplementary Materials

The following supporting information can be downloaded at https://www.mdpi.com/article/10.3390/medicina59122196/s1, Figure S1: Pain trajectory after LRA (numeric pain scale evolution).
