# Supplementary figures and images for: Pain Control for Sickle Cell Crisis, a Novel Approach? A Retrospective Study
Source: Medicina (Kaunas). 2023 Dec 18;59(12):2196. doi: 10.3390/medicina59122196 (PMC10744599; doi:10.3390/medicina59122196)

Figure S1. Pain trajectory after LRA (NPS evolution).

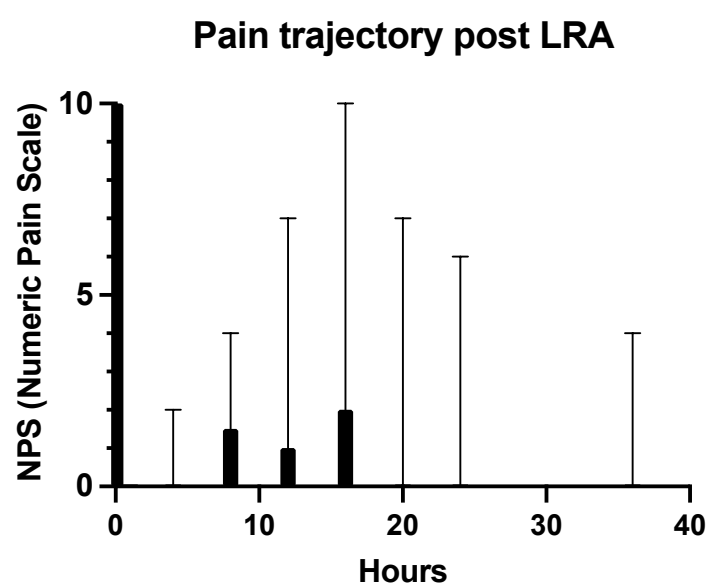

Supplement: Supplementary file 1 [file medicina-59-02196-s001.zip › medicina-2704253-supplementary.pdf]
